# Is early morning flowering an effective trait to minimize heat stress damage during flowering in rice?

**DOI:** 10.1016/j.fcr.2016.11.011

**Published:** 2017-03-01

**Authors:** Raju Bheemanahalli, Rajendran Sathishraj, Muthukumar Manoharan, H.N. Sumanth, Raveendran Muthurajan, Tsutomo Ishimaru, Jagadish S.V. Krishna

**Affiliations:** a International Rice Research Institute (IRRI), DAPO Box 7777, Metro Manila, Philippines; b Department of Agronomy, Kansas State University, Manhattan, KS 66506, USA; c Centre for Plant Molecular Biology and Biotechnology, Tamil Nadu Agricultural University, Coimbatore, Tamil Nadu, India

**Keywords:** Early morning flowering, Heat stress, Rice (*Oryza sativa*), Spikelet sterility

## Abstract

Higher spikelet sterility due to heat stress exposure during flowering in rice is becoming a major threat for sustaining productivity in tropical and sub-tropical regions. Therefore, exploiting and incorporating early morning flowering (EMF) trait into ongoing breeding pipelines could be an effective strategy to minimize the damage. In this study, we have focused on quantifying the time of day of flowering traits such as first spikelet opening time (FSOT) and peak spikelet opening time (PSOT) in a diverse set of cultivars (n = 289) representing major rice growing regions (13 tropical and 20 subtropical countries) over three years (wet season; WS-2012, dry season; DS-2013 and 2014), under field conditions. EMF traits (FSOT and PSOT) and spikelet sterility displayed significant (*p* < 0.001) variations among cultivars, both within and between seasons (WS and DS). Averaged across two dry seasons, the FSOT ranged between 2.35 h and 5.08 h after dawn compared to 3.05 h and 5.50 h during the WS, while, PSOT varied from 3.32 to 6.27 h in DS and from 3.50 to 7.05 h in WS. On average, PSOT was strongly associated with FSOT both in WS (R^2^ = 0.78) and DS (R^2^ = 0.77). A near-isogenic line (IR64 + *qEMF3*) effectively minimized the spikelet sterility by 71% during dry seasons under field conditions compared to 289 tropical and subtropical cultivars. None of the tropical and subtropical originated cultivars possess EMF trait including the popular IR64, thus indicating the usefulness of incorporating this trait to reduce heat stress damage under hotter climate. Our findings provide evidence for the effectiveness of the EMF trait in overcoming heat stress induced sterility under field conditions. Hence, it sounds logical to introgress EMF trait into currently growing popular rice cultivars for improving their resilience to heat stress episodes coinciding with flowering.

## Highlights

• Early-morning flowering (EMF) helps rice plants to escape heat stress damage under field conditions.
• EMF traits and spikelet sterility were estimated from *indica* cultivars originating from 13 tropical and 20 subtropical countries.
• None of the tested 289 cultivars had the EMF trait.
• EMF trait introgressed into popular rice cultivar showed high environmental stability.

## 1 Introduction

Rice is extremely sensitive to short duration heat stress episodes (>35 °C for ≥1 h) coinciding with the reproductive stage, particularly anthesis (Prasad et al., 2006, Jagadish et al., 2007, Jagadish et al., 2008, Jagadish et al., 2010, Sathishraj et al., 2015). Additionally, temperatures above 38 °C occurring even an hour after anthesis had minimal impact on spikelet fertility (Yoshida et al., 1981, Jagadish et al., 2007). Climate models have, with greater certainty indicated increased frequency of hotter days with temperatures above known critical threshold (>33 °C; Jagadish et al., 2007) to coincide with the flowering stage in major rice growing regions (Wassmann et al., 2009, Gourdji et al., 2013, Teixeira et al., 2013, Bheemanahalli et al., 2016). Along similar lines, heat stress induced yield losses in many rice producing regions of China (Li et al., 2004), Japan (Hasegawa et al., 2009), and in other parts of tropical and subtropical regions (Ishimaru et al., 2015) have been reported more frequently over the last decade.

Rice plants possess different mechanisms such as true tolerance (Jagadish et al., 2010) and avoidance through transpiration cooling (Julia and Dingkuhn, 2013) to overcome heat stress inducing damage under field conditions. Recently, the time of day of flowering (Julia and Dingkuhn, 2012) or the early morning flowering (EMF) trait has been demonstrated to significantly reduce heat stress damage by employing an escaping mechanism (Ishimaru et al., 2010, Hirabayashi et al., 2014). During early 1990s, randomly selected rice accessions representing different latitudes and altitudes showed significant variation in start of time of flowering, ranging between 0530 h (*Oryza. eichingeri*) to 2300 h (*O. alta*). However, the peak anthesis (wherein most spikelets flower in a day) in most of the cultivated accessions occurs between 1000 h and 1200 h (Nishiyama and Blanco, 1980, Matsuo and Hoshikawa, 1993, Sheehy et al., 2005). Recent studies have hypothesized that EMF trait could be an effective mechanism to escape heat stress induced spikelet sterility at anthesis by shedding viable pollen on to a receptive stigma during the cooler hours in the morning, to escape sterility inducing temperatures during hours closer to noon. To address this hypothesis, a promising EMF trait or allele has been transferred from wild rice (*O. officinalis*) to mitigate heat stress damage during anthesis (Ishimaru et al., 2010). Further, a stable introgression of EMF quantitative trait locus (QTL) in the *indica* genetic background (IR64 + *qEMF3*) shifted the peak anthesis by ∼2.0 h, compared to the IR64 (recurrent parent), effectively reducing heat stress induced spikelet sterility under growth chamber studies (Hirabayashi et al., 2014). The above study was limited to recording the flowering pattern in the field under sub-tropical humid conditions in the Philippines, but did not test for the agronomic relevance of the trait. The stability of the trait under major rice growing tropical environment and more importantly the ability to alleviate heat stress induced spikelet sterility under field conditions has not been tested so far. There is a strong and active debate that the flowering time is strongly affected by light, humidity and photoperiod (Kobayashi et al., 2010). Therefore, (i) ensuring a stable phenotype or allele expressing EMF in tropical dry environment to complement its performance under sub-tropical humid conditions (Hirabayashi et al., 2014) and (ii) determining its effectiveness to reduce heat stress inducing sterility under field conditions, will help the trait to be readily taken up by breeding programs across major rice growing regions.

Although the agronomic benefit of the trait has been envisaged, there are no systematic observations on the start and peak anthesis involving a larger set of *indica* species (cultivars) originating from different rice growing regions (countries) across the world, to explore the presence/absence of the EMF trait. Hence, three independent experiments (one in wet season 2012 and two in dry seasons 2013 and 2014) were conducted using a set of 289 diverse *indica* cultivars to (i) document the start and time of day of peak flowering among 289 cultivars between wet and dry seasons and (ii) determine the effectiveness of the EMF trait in sterility inducing environment under field conditions, compared with the 289 *indica* cultivars.

## 2 Materials and methods

### 2.1 Crop husbandry

Three independent field experiments were conducted at Paddy Breeding Station (11° N and longitude of 77° E, 426.7 masl), Tamil Nadu Agriculture University (TNAU), Coimbatore, Tamil Nadu, India. A diverse set of 289 cultivars collected from GRISP Global Rice Phenotyping Network (http://ricephenonetwork.irri.org) were phenotyped for flowering traits such as first spikelet opening time (FSOT) and peak spikelet opening time (PSOT) under fully irrigated conditions in one wet season (WS-2012) and two dry seasons (DS- 2013 and 2014). In DS-2015, near isogenic line (IR64 + *qEMF3*) along with the recurrent parent (IR64) were phenotyped for flowering pattern and spikelet sterility under field conditions for assessing the effectiveness and stability of EMF trait in tropical environment.

The soil of the experimental site was clay loamy with pH = ∼7.95, organic matter = 0.42 (%), bulk density of 1.17 (cc/g), extractable P = ∼4.5 (ppm) and exchangeable K = 0.8 (meq/100 g). 21-day-old seedlings of each cultivar were transplanted on a plot area of 3.36 m^2^ with a spacing of 20 × 20 cm (one seedling per hill) as described in Sathishraj et al. (2015). Across experiments, plants received 150 nitrogen (N, kg ha^−1^), 50 potassium (K, kg ha^−1^), 50 phosphorus (P, kg ha^−1^). P was applied as basal dose, N was applied in three splits at tillering (50 kg ha^−1^), active panicle initiation (50 kg ha^−1^) and at heading stages (50 kg ha^−1^). K was spilt applied equally at basal (16.6 kg ha^−1^), panicle initiation (16.7 kg ha^−1^) and at heading (16.7 kg ha^−1^). Details of nursery, crop establishment and fertilizer application was maintained across experiments as reported in Sathishraj et al. (2015). In all three experiments meteorological data were recorded every 15 min using Vantage Pro2™ Davis Automatic Weather Station Instruments, USA, placed besides the experimental plot.

### 2.2 Flowering pattern observations

At heading stage 5 uniform primary tillers (one per plant) were tagged randomly in the middle of the plot (excluding border hills) with least disturbance. On sunny days, flowering pattern observations such as first spikelet opening time (FSOT, the time when first spikelet lemma and palea opens on a given flowering day) and peak spikelet opening time (PSOT, the time when maximum number of spikelets opened) were visually observed from 6.30 to 14.00 (Indian Standard Time, IST) at 15 min interval over three days. To record these traits, cultivars were grouped based on phenology (pedigree information) and by first spikelet opening time data collected on the first day of flowering as reference, by two different observers. Additionally, three different observers recorded the FSOT (example from 8.00 am, based on knowledge on FSOT from Day1) by focusing on tagged main tiller panicles, with either of the observer visiting the same set of plants within 15 min due to a roster method that facilitated higher synchrony among observers using digital calibrated watch. Further, to assess the effectiveness of EMF trait in mitigating high temperature inducing spikelet sterility under field condition, and stable expression under tropical environment, the EMF near isogenic line (NIL, IR64 + *qEMF3*) and its recurrent parent (IR64) were phenotyped for flowering pattern and spikelet sterility in Tamil Nadu, India (DS-2015). Time of dawn, used as a baseline to compare the time of day of flowering across the diverse panel was recorded using weather station Vantage Pro2™ Davis Automatic Weather Station Instruments, USA.

### 2.3 Statistical analyses

Flowering pattern (FSOT and PSOT) data collected across experiments (WS-2012, and DS-2013 and 2014) were normalized taking dawn as the reference. The cultivars used in the study are highly diverse in phenology and in response to photoperiod, which can influence flowering between wet season (kharif) and dry season (summer) in the sub-tropical Tamil Nadu, and hence the need to normalize. Further, the panel of 289 cultivars was classified based on the country of origin and response interpreted based on cumulative average of the trait for cultivars from each country. FSOT and PSOT were analyzed using Genstat (Genstat 17th Rothamsted Experimental Station, Harpenden, UK) and significant differences between means were compared by the least significant difference (LSD) test at the 0.05 probability level.

## 3 Results

### 3.1 Variation in FSOT and PSOT

**Fig. 1:**
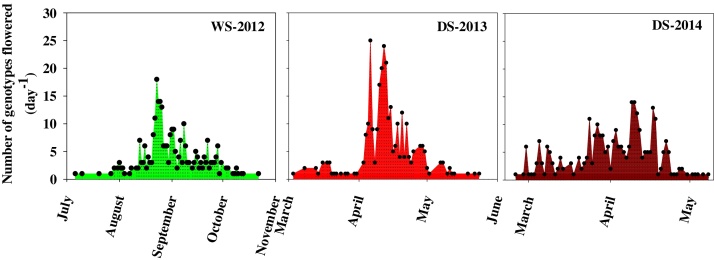
Temporal spread in first spikelet opening time (FSOT) among 289 cultivars in any given day under normal (wet season-WS) and hotter climatic (dry season-DS) conditions in Tamil Nadu, India. FSOT and peak spikelet opening time (PSOT), were observed visually from 6.30 to 14.00 (Indian Standard Time, IST) at 15 min interval by focusing on tagged main tiller panicles (for details see materials and methods).

**Fig. 2:**
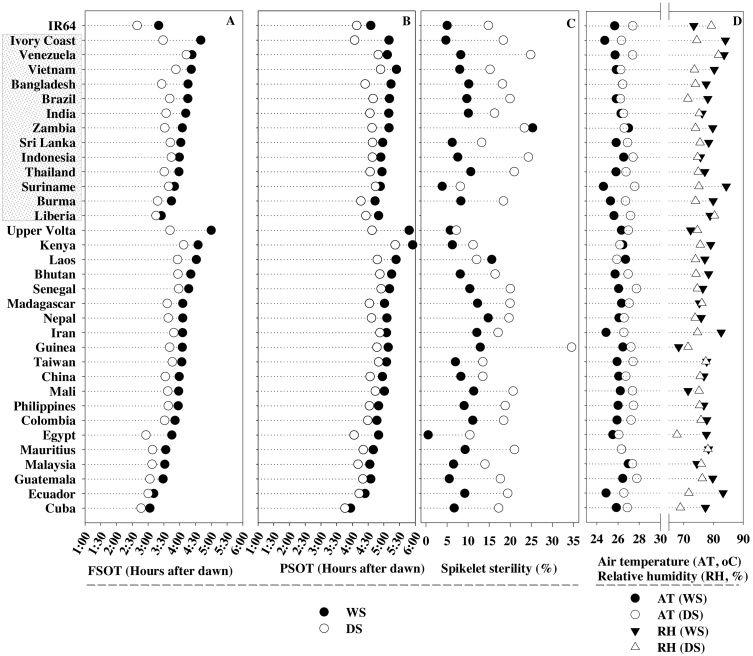
Genetic variability in flowering traits including first spikelet opening time (FSOT; A) peak spikelet opening time (PSOT; B) and percent spikelet sterility (C) among rice cultivars originated from tropical and sub-tropical regions and tested under normal (wet season-WS) and hotter climate (dry season-DS) conditions in Tamil Nadu, India. Cumulative average of air temperature (AT) and relative humidity (RH, %) from dawn to six hours after dawn on the day flowering pattern was measured (D). Countries in and outside the box on Y axis are tropical and sub-tropical regions of origin of the cultivars, respectively. IR64, a popular cultivar is used as a reference and data presented is the average across all cultivars a country, with response of individual cultivar provided in Supplementary Table 1.

Early morning flowering traits such as first spikelet opening time (FSOT) and peak spikelet opening time (PSOT) showed significant genetic variation among cultivars (Supplementary Fig. S1). In the entire panel, the number cultivars showing first spikelet opening in a day varied between 1–18 in WS-2012, 1–25 in DS-2013 and 1–14 in DS2-014 (Fig. 1). Significant shift in flowering pattern (early flowering) was recorded in dry seasons (*p* < 0.001) with higher spikelet sterility compared to wet season (WS), see Fig. 2. Further, cultivars classified based on their country of origin (tropical and subtropical) also displayed significant (*p *< 0.001) variations across experiments, but their mean duration of FSOT, PSOT, and spikelet sterility between cultivars from tropical and subtropical regions was not significant (*p *> 0.05).

Cultivars from different geographic regions took 3.01–5.50 h for first spikelet opening (FSOT) after dawn during WS, whereas in DS it ranged from 2:35 to 5.08 h (see Supplementary Table S1 for individual responses of 289 genotypes). It was noticed that ∼91% cultivars during WS and 99% during DS had their FSOT later than IR64, while during DS only three cultivars from the 289 were either slightly earlier or equal to IR64 (Fig. 2A and Supplementary Table S1). The peak spikelet opening time (PSOT) displayed larger variation in WS (3.50–7.05 h) compared to DS (3.32 h to 6.27 h) (Supplementary Table S1 and Fig. 2B). Similarly, wide variation in PSOT was seen among tropical (3.50–7.05 h in WS and 3.32–6.27 h in DS) and sub-tropical (3.50–6.05 h in WS and 3.35–5.45 h in DS) cultivars (Fig. 2B). On average, PSOT of all the tropical (5.04 and 4.34 h) and subtropical (4.59 and 4.37 h) cultivars were delayed by about 0.25–0.30 h than the popular cultivated IR64 (4.35 h and 4.08 h), in WS and DS, respectively (Fig. 2B). In comparison to IR64, ∼79% and 86% of the cultivars took longer duration to reach PSOT in WS and DS, respectively (Fig. 2B). The mean FSOT and PSOT of IR64 were 3.20 h and 4.35 h in WS, while in the DS these duration were reduced by 0.40 and 0.26 h, respectively (Fig. 2). A strong positive association between FSOT and PSOT were noticed in WS (R^2^ = 0.77, *p* < 0.001; Supplementary Fig. S2A) and DS (R^2^ = 0.78, *p* < 0.001; Supplementary Fig. S2B). Although, shifts in FSOT and PSOT were mostly narrow during DS compared to WS (Fig. 1A and B), spikelet sterility in DS was significantly increased by 49% in tropical (WS −8.8% and DS −17.2%) and 44% in subtropical (WS −9.8% and DS −17.6%) cultivars compared to WS (Fig. 2C). Higher air temperatures (AT) at flowering (from dawn to six hours after dawn) were recorded in DS (24.6 to 28.6 °C) compared to WS (24.1 to 27.5 °C) (Fig. 2D). Spikelet sterility during DS was significantly correlated with AT (r = +0.20, *p* < 0.001; n = 289) but not with RH. On the other hand, spikelet sterility in WS had no significant relationship with either AT or RH.

### 3.2 Effectiveness and environmental stability of EMF trait

For comprehensive understanding of the response, environmental stability and effectiveness of EMF trait across locations, a near-isogenic line (NIL) developed in background of IR64 (IR64 + *qEMF3*) at IRRI (Hirabayashi et al., 2014) was phenotyped and compared to the tropical and subtropical group of cultivars from the diverse panel.

**Fig. 3:**
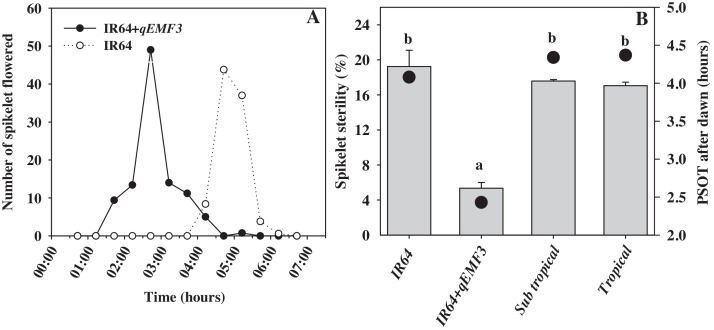
Time of day of flowering (TDF) in IR64 + *qEMF3* and recurent parent (IR64) under field conditions in hot tropical climate. A) To estimate TDF, number of opened spikelets per panicle (five panicles from different plants) were counted at every 15 min interval following Jagadish et al., 2007 procedure. Closed black circles and open circles represent cumulative average number of spikelet’s opened per panicle over five days. B) Effectiveness of early morning flowering in overcoming high temperature inducing spikelet sterility was accounted in DS-2013 and DS-2014 (tropical and subtropical cultivars) and DS-2015 (IR64 + *qEMF3* and recurrent parent, IR64). Black circle symbols is the PSOT. Bars indicate the mean ± standard error. Alphabet with the same letter are non-signifcant at *p* < 0.05.

There were significant differences in time of anthesis and spikelet sterility between IR64 + *qEMF3* and IR64 (Fig. 3A and B) in DS-2015 (Tamil Nadu, India), respectively. FSOT in IR64 + *qEMF3* started 2.30 h prior compared to IR64 (4.13 h after dawn) and reached peak flowering ∼2 h early than IR64 (10.30–11.00 am) (Fig. 3A). The mean PSOT of the IR64 + *qEMF3* was 2.43 h in DS-2015 after dawn, which is significantly (*p* < 0.001) earlier than any other cultivars [289] used in the study (Fig. 2 and 3B). The IR64 + *qEMF3* had ∼ 1.25 h earlier PSOT compared to IR64 (mean of both 2014 and 2015 dry seasons in Tamil Nadu) followed by tropical (1.51 h) and subtropical (1.54 h) cultivars during DS (Fig. 3B). These results demonstrated that IR64 + *qEMF3* effectively induced peak flowering to occur under cooler hours under tropical conditions similar to humid sub-tropics (Hirabayashi et al., 2014), to prevent exposing anthesis to late-morning or early-afternoon hot weather conditions. In IR64 + *qEMF3*, the start and peak spikelet opening occurred earlier in the day and hence resulted in 69–72% lower spikelet sterility (5.35%) compared to IR64 (19.2%, cumulative average of three dry seasons), tropical (17. 1%) and sub-tropical (17.6%) rice cultivars (Fig. 3B). These results provide first evidence of the effectiveness and stable expression of the EMF trait in maintaining spikelet fertility across different rice growing environments under field conditions, highlighting the need to introgress the trait for mitigating heat stress induced spikelet sterility.

## 4 Discussion

To minimize heat stress induced reduction in spikelet fertility, rice plants employ either escape, avoidance, true tolerance or a combination of these mechanisms under field conditions (Satake and Yoshida, 1978). Heat escape through early-morning flowering (Ishimaru et al., 2010, Hirabayashi et al., 2014), heat avoidance through transpiration cooling (Julia and Dingkuhn, 2013) and heat tolerance by altering cellular metabolites to increase resilience during reproductive processes (Jagadish et al., 2010) has been documented. Although, these mechanisms are well known, it is only recently that EMF trait identified by Sheehy et al., 2005 was characterized and mapped and genomic regions responsible for this phenotypic variation were identified (Ishimaru et al., 2010, Thanh et al., 2010, Hirabayashi et al., 2014). However, till date, there has been no attempt to assess the flowering pattern (FSOT and PSOT) among diverse set of germplasm to ascertain the agronomic importance of this trait. This is the first attempt to record key flowering pattern traits involving 289 cultivars originating from 33 countries (13 tropical and 20 subtropical) over three years (wet season-2012, dry season-2013 and 2014) under fully irrigated field conditions (Supplementary Fig. S1 and Fig. 2). Variations in time of day of spikelet opening (FSOT and PSOT) was observed among the cultivars in the panel (Fig. 2 and Supplementary Table S1), but, none of the cultivars possessed EMF trait as seen in O*. officinalis* (Ishimaru et al., 2010), Nanjing 11 + *qEMF3* and IR64 + *qEMF3* (Hirabayashi et al., 2014). Further, EMF allele introgressed NIL (IR64 + *qEMF3*) expressed true early morning trait with minimum G x E interaction as the trait in IR64 background had similar flowering pattern under humid sub-tropical (Hirabayashi et al., 2014) and dry tropical environment (TNAU, current study). Interestingly this NIL recorded lower spikelet sterility compared to *indica* cultivars and its recurrent parent in dry season (Fig. 3B), a first report of the effectiveness of the traits in overcoming heat stress induced sterility under field conditions. Origin of cultivar (region) was not associated with changes in EMF (FSOT and PSOT) and spikelet sterility and a similar non-significant relationship between latitude or altitude and time of day of flowering of the cultivated cultivars was reported by Sheehy et al., 2005. Although, the *indica* rice panel investigated did not possess EMF trait, there are few cultivars which have the ability to maintain spikelet sterility similar to IR64 + *qEMF3* (Supplementary Table S1**)**. These cultivars would most probably have other adaptive mechanisms for overcoming heat stress effect at anthesis such as avoidance or tolerance or a combination of both under field conditions. These results provide convincing evidence that the EMF allele (*qEMF3*) introgressed in to Nanjing 11 and IR64 is not present among the cultivars studied, possibly excluded during breeding efforts focused on increasing yield or due to a narrow genetic diversity incorporated into current breeding programs. The EMF allele introgressed into popular variety (IR64 + *EMF3*) reached peak flowering ∼2 h prior to recurrent parent (IR64), hence recording higher seed-set due to higher spikelet fertility even under hotter climatic conditions (Fig. 3B). This indicates the effectiveness and stable performance of the trait across diverse environments providing confidence to initiate trait based breeding program in target countries to minimize heat stress induced spikelet sterility.

Flowering has been noted as the most sensitive stage to heat stress, and prevailing air temperatures during anthesis (flower opening) have been significantly linked to reproductive success or failure (Yoshida et al., 1981, Jagadish et al., 2007). Introgressing EMF traits or allele could help peak flowering to occur closer to dawn thereby helping flowering spikelets to escape late-morning and early-afternoon heat stress (Jagadish et al., 2015), particularly under tropical and subtropical rice growing regions that are identified with greater vulnerability (Wassmann et al., 2009). This promising trait will facilitate the heat sensitive physiological processes including anther dehiscence, pollination (Matsui et al., 2007, Jagadish et al., 2010), pollen germination and pollen tube growth and even fertilization to occur under non-stress cooler conditions. Although underpinning the mechanisms associated with EMF traits across different environmental conditions using advanced physiological and genomic tools is an interesting future line of work, the trait already provides significant promise to mitigate heat stress damage and needs to be integrated into current ongoing breeding programs. With the predicted increase in global temperatures, escaping heat stress by incorporating EMF will further increase the advantage with greater increase in severity of stress.
