# Supplementary material for: Is early morning flowering an effective trait to minimize heat stress damage during flowering in rice?
Source: Field Crops Res. 2017 Mar 1;203:238–42. doi: 10.1016/j.fcr.2016.11.011 (PMC5310116; doi:10.1016/j.fcr.2016.11.011)
Supplement: Supplementary file 1 [file mmc1.docx]

**Supplementary Figure. S1:** Frequency distributions of FSOT and PSOT for the 289 rice cultivars in wet season (WS) and dry season (DS) under field conditions. A set diverse 289 cultivars were phenotyped for first spikelet opening time (FSOT) and peak spikelet opening time (PSOT) under fully irrigated conditions in one wet season (WS-2012) and two dry seasons ( DS- 2013 and 2014). A pooled mean across both 2013 and 2014 dry seasons was used for the analysis.

**Supplementary Figure. S2.** Relationship between first spikelet opening time (FSOT) and peak spikelet opening time (PSOT) among diverse rice accessions across wet season (WS, A) and dry season (DS, B). *** - *p* < 0.001. N- number of cultivars.
